# Static Ice Pressure Measuring System Based on Fiber Loop Ring-Down Spectroscopy and FPGA

**DOI:** 10.3390/s20205927

**Published:** 2020-10-20

**Authors:** Xiao Deng, Dingrui Wang, Lipeng Pan, Li Zhang, Jun Zhang, Xinshuo Lu, Chao Du, Lin Zhang

**Affiliations:** 1 College of Physics and Optoelectronics, Taiyuan University of Technology, Taiyuan 030024, China; wangdingrui0863@link.tyut.edu.cn (D.W.); panlipeng0575@link.tyut.edu.cn (L.P.); zhangli06@tyut.edu.cn (L.Z.); zhangjun0844@link.tyut.edu.cn (J.Z.); luxinshuo0743@link.tyut.edu.cn (X.L.); duchao@tyut.edu.cn (C.D.); 2 Key Laboratory of Advanced Transducers and Intelligent Control System, Ministry of Education and Shanxi Province, Taiyuan 030024, China; 3 College of Architecture, Taiyuan University of Technology, Taiyuan 030024, China; zhanglin@tyut.edu.cn

**Keywords:** static ice pressure, fiber loop ring-down spectroscopy, FPGA pulse modulation, continuous detection

## Abstract

Hydraulic engineering built in the cold region, such as reservoirs and hydropower stations, is often threatened by static ice pressure from nature. Therefore, it is of vital significance to research the pressure variation in the growth and melting processes of the ice layer for the design and protection of hydraulic structures in cold regions. This paper introduces an optical fiber sensor system based on the fiber loop ring-down spectroscopy technology and field-programmable gate array (FPGA) pulse modulation technology. An electro-optic modulation scheme that relied on FPGA to generate optical pulses with adjustable pulse width and period is proposed, which is more suitable for the in-situ observation. In addition, the temperature stability and repeatability of the system are also discussed. This system was applied to the real-time detection of static ice pressure on the sidewall and bottom of the polyvinyl chloride (PVC) pipe during the ice growth and melting processes. The results indicate that the system has favorable stability and sensitivity, and the relationship obtained between the static ice pressure and temperature could provide some references for the field application in the future.

## 1. Introduction

### 1.1. Background

In cold regions, icing often occurs on the water surface in rivers, lakes, and reservoirs. Due to the intimate contact with the ice layer, various hydraulic structures located here, such as oil platforms, bridge piers, and abutments, will be subjected to the pressure exerted by the ice layer [1,2]. During the winter season, if the impact of drift ice collisions is not considered, the ice thickness and static ice pressure in still water will increase as the cumulative negative temperature increases [3,4]. This state will be broken when the temperature changes drastically. Especially in spring, when the ice cover absorbs more heat, its volume expands rapidly and will threaten the safety of hydraulic structures such as the earth dam slope, intake tower, and bridge pier [5,6,7,8,9]. Therefore, when designing hydraulic facilities, the ice load of the whole cycle in winter must be considered.

### 1.2. Research Method of Static Ice Pressure

There are three main methods to research the ice load of structures. The model test method usually uses an ice pool to simulate the actual situation of the field environment and measures the mechanical properties of ice under laboratory conditions [10,11]. However, the disadvantage of this method is that low-temperature experimental conditions need to be implemented in a special environment that can be controlled, thus the investment is high. In addition, the simulated environment cannot precisely reproduce various parameters in the site environment. Therefore, this method needs to expand in both depth and breadth. The theory and calculation methods are not perfect, and there are still many problems in the exploratory stage. Thus, the experimental results are only for reference [12,13,14]. The numerical simulation method uses algorithms and software to determine the key parameters of the ice cover through hydrological and meteorological data. Then a mathematical model is established to determine the static ice load [15,16]. The advantage is that the known factors can be input into the model as parameters, but its theoretical calculations are complex, boundary conditions are not easy to measure, accuracy is low and real-time capability is bad [17,18]. The in-situ observation method needs to deploy some pressure sensors (such as biaxial stress sensors [19] and strain gauges [20,21]) in advance at the hydraulic facility, and the pressure variation data inside the ice layer can be obtained through manual timing observation and recording. It can be summarized as a simple and effective prediction model combined with other environmental parameters. Compared with the model test method and numerical simulation method, the in-situ observation method is closer to the spot practical situation, which has a better practical reference. Therefore, the method of in-situ observation has been widely adopted. For example, 11 biaxial stress sensors were deployed to the La Gabelle reservoir in south-central Quebec, Canada, and ice stress data for two winter seasons were obtained [22]. By equipping the steel gate with multiple vibrating wire strain gauges, the ice pressure at various locations on the structure over 100 days was collected [23]. Using strain gauges, four ice load measurements were performed in the Beaufort Sea, and the relationship between ice pressure and the area was obtained [24]. However, due to the limitation of the volume of the sensing unit, the traditional embedded observation method will damage the structure of the ice cover during deployment. It cannot accurately reflect the actual force of the medium, nor can it detect the mechanical properties of the ice sheet during the melting process.

Therefore, in the face of the influence of low temperature and complex environment and the constraints of ice pressure measurement technology of field environment, it is necessary to explore a new method for continuous static ice pressure measurement applied to the field environment.

### 1.3. Optical Fiber Sensor for Static Ice Pressure Detection

In recent years, the fiber sensor has attracted extensive attention because of its advantages such as miniaturization, no power supply, fast response speed, strong corrosion-resistance, and anti-electromagnetic interference [25,26,27,28,29]. Some work has also been carried out on ice pressure detection. A Y-type optical fiber sensor based on the principle of light intensity modulation realized continuous automatic detection of static ice pressure [30]. It was also proposed that fiber bragg grating (FBG) can be used to study the thermodynamic properties of saline ice, the structural deformation of ice material under external mechanical impact, and ice load on bridge piers [31,32,33]. The Brillouin optical time-domain analysis sensor was installed in the ice block and ice beam to monitor strain and ice structure damage characteristics [34]. For some harsh field environments (such as low temperature and icing environment), these methods still have some shortcomings. First, most optical fiber sensors are affected by the input light intensity. The fluctuation of the input light will produce errors in the measurement results. Second, there are requirements for integration and automation of the measurement system in harsh conditions, but the current system cannot meet these requirements to achieve automatic continuous detection. Therefore, a monitoring method that can overcome the complex structure of traditional fiber sensors contributes to actual field applications. Fiber loop ring-down spectroscopy (FLRDS) technology has the advantages of simple structure and good reliability, which has broad prospects in pressure measurement [35,36,37]. Wang et al. [38] first applied FLRDS technology to pressure sensing. Jiang et al. [39] reported that pressure measurements in the range of 0 to 32.5 MPa were achieved using FLRDS pressure sensors.Gao et al. [40] proposed a four-channel fiber loop ring-down pressure sensor that can simultaneously perform separate pressure measurements on four channels. Nevertheless, research on the application of FLRDS technology at low temperature is rarely reported. In addition, the light source module in the traditional FLRDS system is not conducive to integration and application in the field environment. Relying on the field-programmable gate array (FPGA) electro-optical modulation method, the light source parameters can be adjusted according to the site’s actual situation, which is more suitable for the use of field environment.

In this paper, according to the requirements of FLRDS technology for the pulse width and repeatability of the optical pulse, we first rely on the programmable logic characteristics of the FPGA to generate the electrical pulse. Then the electric pulse is injected into the electro-optic modulator for intensity modulation, and finally a stable optical pulse is generated. On this basis, combined with the FLRDS technology, a novel measuring system based on FPGA was built. The measurement results of the growth and melting processes on the sidewall and bottom of the ice layer are also described.

The main contributions of this work are as follows:

1. An FPGA-based optical pulse generation system is designed, which can generate light pulses with controllable pulse width and period and is conducive to realizing field applications.
2. For measuring the static ice pressure during the ice growth and melting processes, the exiting micro bend sensor is optimized, and 4 auxiliary fibers are added to expand the range of the sensor while ensuring the sensitivity.
3. The static ice pressure on the sidewall and bottom of the ice cover was studied, respectively. The static ice pressure changes at different depths on the sidewall of the ice cover are obtained. Meanwhile, the relationship between the static ice pressure on the sidewall and the bottom of the ice cover are obtained.

## 2. Materials and Methods

**Figure 1:**
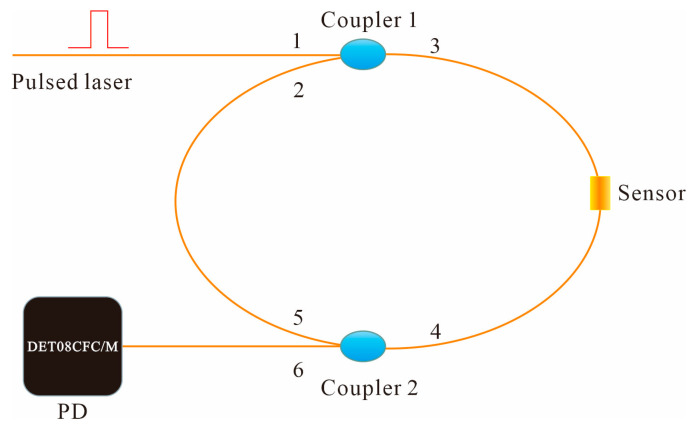
Measuring principle of fiber loop ring-down spectroscopy (FLRDS).

The measuring principle of static ice pressure based on the fiber loop ring-down spectroscopy is shown in Figure 1.

The FLRDS resonator was composed of 2 identical couplers with a high coupling ratio and a section of single-mode optical fiber. The total length of the fiber loop was 250 m, of which the length of the sensing area was 70 mm. When the pulse laser was injected into the optical resonator through the port 1 of coupler 1, the pulse laser will pass through the fiber loop circularly until the signal disappears, caused by the optical loss. During every circulation, a fraction of the optical pulse will emit from the port 6 of coupler 2. The periodic trains of emitted signals were detected by a photodetector (PD).

The measuring principle of FLRDS complies with the Beer-Lambert law, and the output optical pulse signals exhibit the characteristic of exponential decay, which can be defined as follows:

(1) $$I=I_{0}\exp\left(-cAt/nL\right)$$

where *I* is the output light intensity of loop cavity at time *t*, $\;I_{0}$ is the initial intensity of the optical pulse, *c* is the speed of light, *A* is the inherent loss of fiber resonator including absorption loss, insertion loss, and fiber-to-fiber insertion loss, *n* is the refractive index of the fiber, and *L* is the total fiber length of fiber resonator.

Ring-down time caused by the inherent loss *A* could be defined as the time when the light intensity decreases to 1/e of the initial light intensity $I_{0}$. Thus, the ring-down time $\tau_{0}$ can be expressed as follows:

(2) $$\tau_{0}=\frac{nL}{cA}$$

When the pressure *P* acts on the sensing unit, it will induce the additional loss *B* and the new ring-down time *τ* that can be defined as:

(3) $$\tau =\frac{nL}{c\left(A+B\right)}$$

Therefore, the pressure on the sensor unit can be expressed by:

(4) $$\frac{1}{\tau }-\frac{1}{\tau_{0}}=kP$$

where *k* is the related coefficient and defined as a constant. We could obtain the pressure value by the ring-down time before and after a measurement. 

## 3. The Detection System of Static Ice Pressure

### 3.1. Optical Pulse Modulation-Based FPGA

**Figure 2:**
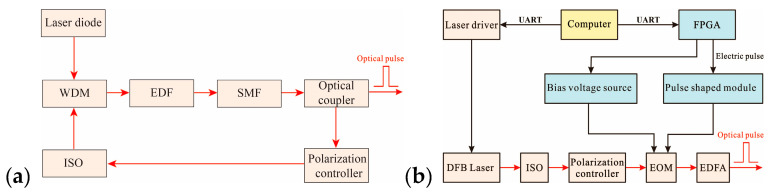
Schematic diagram of the optical pulse modulation scheme; (**a**) traditional optical pulse modulation scheme; (**b**) Field-programmable gate array (FPGA)-based optical pulse modulation scheme.

The pulsed light source of the traditional FLRDS system consisted of a laser diode, a wavelength-division-multiplexer (WDM), an erbium-doped fiber (EDF), a single-mode fiber (SMF), a polarization controller, and an optical isolator (ISO), as shown in Figure 2a.

The traditional system cannot adjust the light source parameters according to the field environment and was complicated, which increased the cost of the entire system and caused difficulties for the integration and application in the field environment.

We proposed a scheme for the generation of optical pulses based on the FPGA. The system composition is shown in Figure 2b. A narrow linewidth distributed feedback (DFB) laser with a central wavelength of 1550 nm was used as a light source. After the continuous light of DFB was injected into the ISO, the light outputted from the ISO was transferred into the electro-optic modulator (EOM) through the polarization controller. The polarization controller before the EOM was employed to align the light polarization to the E-O crystal of the modulator, maximizing its extinction ratio. At the same time, under the combined action of the electric pulse shaped and the bias voltage source, the continuous light inside the EOM was modulated into the optical pulse with the same pulse width and period according to the electric pulse. However, the optical pulse modulated by EOM was so weak that it could not be directly used in our detection system. Thus, we choose an Erbium-doped fiber amplifier (EDFA) to amplify the power of the optical pulse. The pulse width and period of the light pulse can be controlled by FPGA instruction, which was conducive to field environment use.

**Figure 3:**
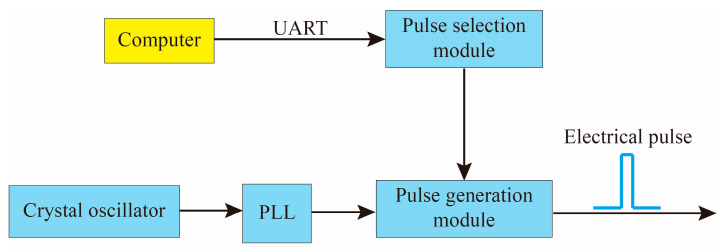
Structure diagram of pulse signal source based on phase-locked loop (PLL).

Light pulses need to be generated by electrical pulse modulation. We designed a pulse signal source based on phase-locked loop (PLL) technology to meet the electrical pulse requirements of the FLRDS system, as shown in Figure 3.

A 50 MHz crystal oscillator was used as the system clock of the FPGA, which was appropriately multiplied by the PLL and then the input to the pulse signal generation module. According to the length of the actual optical fiber loop ring-down cavity, the computer sent out the corresponding command signal to control the pulse selection module to output pulse signals of different widths and periods.

The electric pulse width can be adjusted by:

(5) $$d=\frac{K}{rF_{clk}}$$

where *d* is the pulse width, *F_clk_* is the system clock, *r* is the frequency multiplication coefficient of the PLL, and *K* is frequency division coefficient, which is a positive integer.

Besides, it can be approximated that the width of the electric pulse is equal to the width of the light pulse, and the requirements for the width of the electric pulse can be defined as follows:

(6) $$d\leq \frac{nL}{c}$$

where *n* is the refractive index of the fiber, *L* is the total fiber length of fiber resonator, and *c* is the speed of light. That is, the width of the electrical pulse must be less than the time required for the pulsed light to transmit one circle in the fiber loop. Otherwise, the optical pulse will cause interference phenomenon in the optical fiber ring, which will affect the stability and accuracy of the system.

The pulse repetition frequency is related to the number of pulse ring-down, which can be defined as follows:

(7) $$T>\lambda \frac{nL}{c}$$

where *T* is the pulse period, and λ is the number of ring-down of the pulse in the fiber loop. That is, the pulse period must be greater than the propagation time of the optical pulse in the fiber loop.

**Figure 4:**
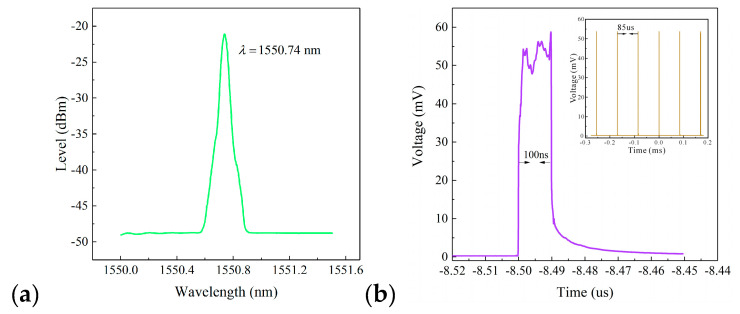
Characteristics of the generated optical pulse; (**a**) the pulse spectrum; (**b**) the pulse width and the repetition period.

Characteristics of the optical pulse-modulated of the detection system are shown in Figure 4.

We can see that the center wavelength of the output spectrum of the optical pulse was 1550.74 nm (Figure 4a), and the pulse width and the repetition period of pulse sequence collected by the oscilloscope was 100 ns and 85 μs, respectively (Figure 4b). 

### 3.2. Experimental Setup

**Figure 5:**
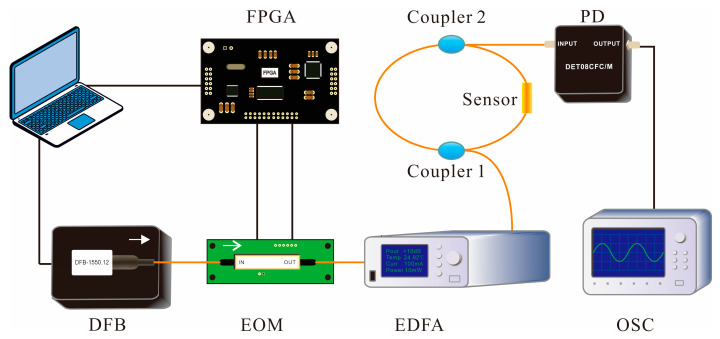
Schematic diagram of the static ice pressure detection system.

The schematic diagram of the detection system for static ice pressure is shown in Figure 5.

It mainly consisted of the optical pulse source, FLRDS resonator integrated sensing unit, and data acquisition device. The FLRDS resonator was composed of two identical 2 × 1 fiber couplers with a coupling ratio of 90:10, the single-mode fiber of 250 m, and a sensing unit designed by micro bending theory. The data acquisition device included an InGaAs photodetector (DET08CFC/M, Thorlabs, NJ, USA) and an oscilloscope (OSC, MSO-X3102T, Agilent, California, USA). The 10% exported optical pulse taken from the coupler 2 was converted into the electrical signal by the photodetector, and the electrical signals were collected by the oscilloscope and stored in the SD card.

The fiber micro-bend sensor was first proposed by J.N. Fields and J.H. Cole in 1980 [41]. They introduced a fiber micro-bend hydrophone, which can detect a minimum signal of 95 dB/μPa. Due to mechanical disturbance, the optical fiber was slightly bent, thereby redistributing the optical energy contained in the different modes in the optical fiber. The more the fiber was bent, the more the light energy was occupied by the radiation mode generated in the fiber, and the greater the optical loss. Using this feature, the optical fiber was clamped between 2 deformed plates with a bending structure to form a fiber micro-bend sensor. At present, micro-bend sensors have a wide range of applications in the measurement of refractive index [42], strain [43], and displacement [44].

**Figure 6:**
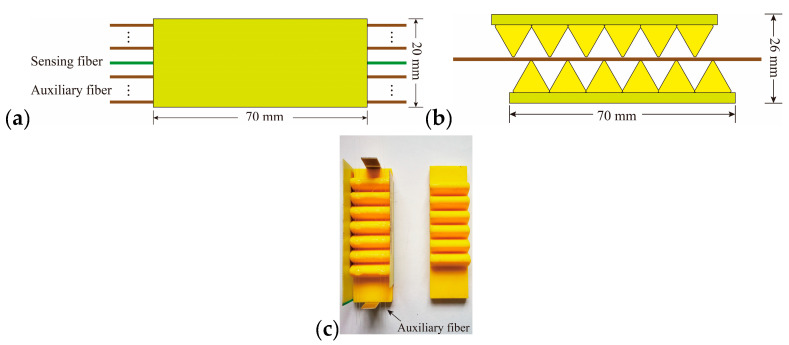
Schematic diagram and picture of the optical pressure sensor; (**a**) top view of the pressure sensor; (**b**) front view of the pressure sensor; (**c**) picture of the pressure sensor.

The pressure sensor with a single sensing fiber was sensitive, but the measurement range was small. Moreover, in the growth and melting processes of ice, the static ice pressure varied widely. Especially in the melting process of ice, excessive static ice pressure will cause the sensing fiber to break. In order to avoid this situation, the method of adding auxiliary fiber was used in the experiment to increase the measuring range of the sensor while ensuring the sensitivity, as shown in Figure 6.

As the number of auxiliary fiber increase, the measurement range of the sensor will gradually increase, and the sensitivity will gradually decrease. In order to increase the measurement range and ensure that the sensitivity is within an acceptable range, we used 4 auxiliary fibers. In order to ensure the force balance of the sensor, we used the shadowless glue used for fiber bonding to fix the auxiliary fibers thus that they were symmetrically distributed on the sawteeth. When the static ice pressure was applied to the sensor, the pulsed light caused loss at the micro bend of the optical fiber, causing the change of the ring-down time. According to Equation (4), the functional relationship between the ring-down time and the pressure can be obtained.

In order to ensure the sensor is uniformly forced and the upper and lower sawteeth will not slip relatively, the lower sawteeth were fixed thus that the upper sawteeth only moved in the direction of the force and would not be displaced in the non-forced direction.

**Figure 7:**
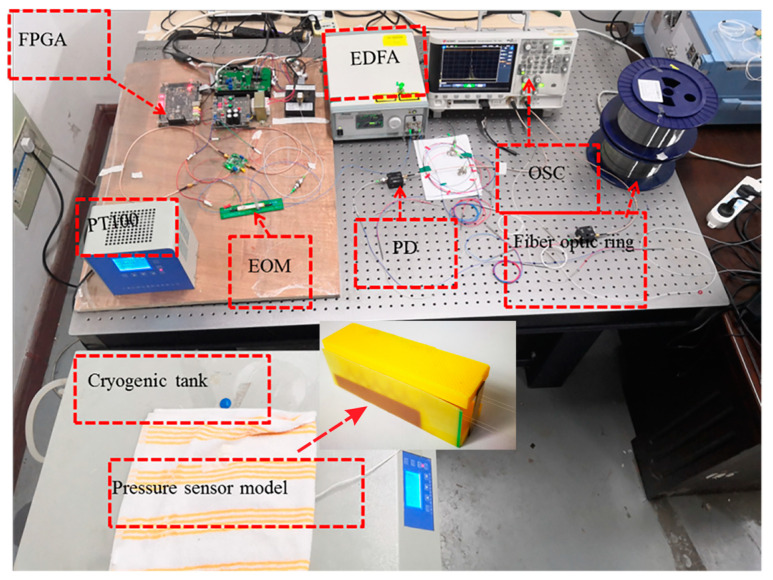
The picture of static ice pressure sensor system.

Figure 7 shows the experimental site of static ice pressure sensing in the laboratory. In the experiment, a cryogenic tank was applied to simulate a natural icing environment.

The cryogenic tank (BILON-W-506S, BILON, Shanghai, China) had an adjustable temperature range of −50 ℃ to 90 ℃, which can simulate the growth and melting processes of river ice in winter. In addition, a digital thermometer with a PT100 sensor probe that came with the cryogenic tank was used to measure the temperature change inside the ice in real-time.

For calibration and repeatability testing of pressure sensors on the sidewall and bottom, the fiber optic pressure sensor was fixed on the tension frame, which was placed on the optical platform to ensure horizontal. Applying pressure to the sensor through a pressure gauge (HP-500, SHSCYQ, Shanghai, China, the load resolution was 0.1 N, the measurement range was 0 to 500 N) to make the sensor evenly stressed. The end of the pressure gauge was connected to a spiral micrometer to control the applied pressure accurately.

When the pressure was applied to test the repeatability of the sidewall and bottom pressure sensors, the pressure duration was 10 min, and the corresponding ring-down time was measured at this time. The time interval of each measurement was 1 min, and 10 sets of data were measured in 10 min. The pressure was then not applied to the sensor for 10 min to perform the same measurement, and this cycle was repeated 5 times.

**Figure 8:**
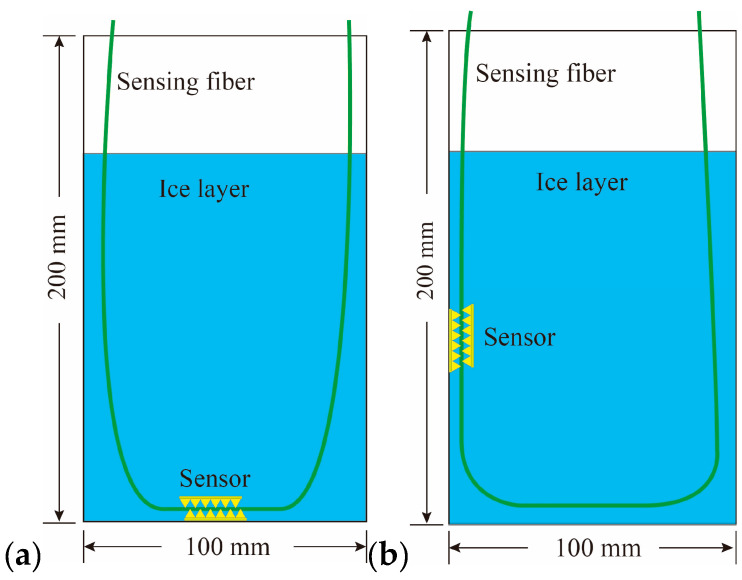
The sensor layout diagram; (**a**) measuring the static ice pressure on the bottom; (**b**) measuring the static ice pressure on the sidewall.

The sensor layout diagram is shown in Figure 8.

Since the ice expands during the processes of growth and melting, it will exert pressure on the pipe wall. In order to ensure that the wall of the barrel will not be deformed, the PVC with higher strength was used as the pipe material. The PVC pipe was 10 cm in diameter and 20 cm in height. When measuring the static ice pressure at the bottom, the sensor was placed at the water bottom. Furthermore, when measuring the static ice pressure on the sidewall, the sensor was arranged at depths of 10 cm, 13.5 cm, and 16.5 cm to explore the relationship between different depths and static ice pressure, respectively.

## 4. Experimental Results and Discussion

### 4.1. Temperature Stability

**Figure 9:**
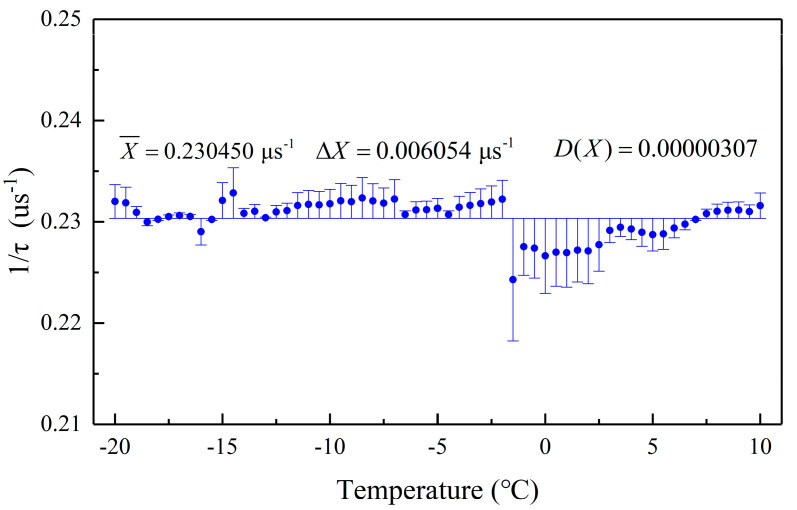
Stability test results of temperature ranged from −20 ℃ to 10 ℃.

During the experiment, the static ice pressure was huge, and the sensor will withstand pressure for a long time. In order to explore the recovery performance of the optical fiber pressure sensor, we first tested the temperature stability of the sensor. Figure 9 shows that when the temperature ranges from −20 ℃ to 10 ℃, the ring-down time of the optical fiber sensor is almost unchanged, and the variance of its reciprocal is 0.00000307, indicating that the temperature has little effect on the sensor.

### 4.2. Static Ice Pressure to the Sidewall of the Ice Tank

**Figure 10:**
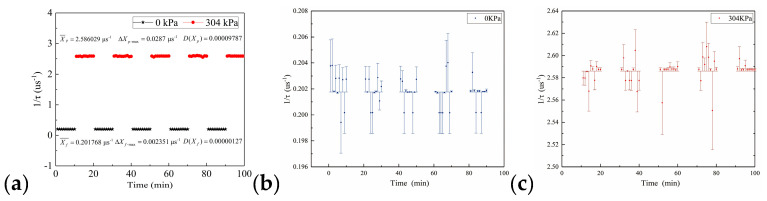
Repeatability test results of pressure sensor on the sidewall; (**a**) the ring-down time curve when 304 kPa is loaded and not loaded on the sensor; (**b**) the error bar of 1/*τ* response when 304 kPa is not loaded on the sensor; (**c**) the error bar of 1/*τ* response when 304 kPa is loaded on the sensor.

By measuring the 1/*τ* response of the 304 kPa pressure loaded and unloaded on the sensor, the repeatability of the sidewall static ice pressure was investigated, which is presented in Figure 10a.

When the pressure was not applied to the sensor (the pressure is 0 kPa), the average value of the reciprocal of the ring-down parameter was 0.201768 μs^−1^, whereas the maximum error was 0.002351 μs^−1^, and the variance was 0.00000127, as shown in Figure 10b. Additionally, when the pressure was 304 kPa, the average value of the reciprocal of the ring-down parameter was 2.586029 μs^−1^, whereas the maximum error was 0.028700 μs^−1^, the variance was 0.00009787, as shown in Figure 10c. The results showed that the maximum error was within the acceptable range, and the test results were relatively stable, which can meet the measurement requirements of the sidewall static ice pressure. At the same time, when 304 kPa was loaded on the sensor, the 1/*τ* response increased immediately. Otherwise, when the pressure was removed, the 1/*τ* response dropped sharply. The results indicated that the pressure sensor was sensitive to pressure and had good repeatability.

**Figure 11:**
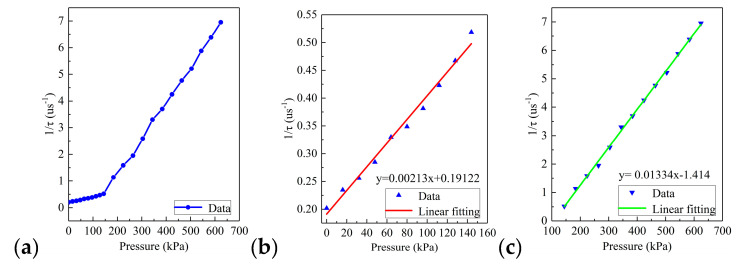
The relationship between the ring-down time and static ice pressure on the sidewall; (**a**) ring-down time-pressure curve of the overall range of sensor; (**b**) calibration curve of the low-pressure area; (**c**) calibration curve of the high-pressure area.

We further studied the relationship between different pressure and ring-down time, as shown in Figure 11.

The ring-down time decreased as the pressure increased. From Figure 11a, it can be found that the calibration result was not a single linear relationship, and the sensor needed to be calibrated sectionally. Therefore, Figure 11b,c shows the calibration of the low-pressure area and high-pressure area of the sensor, respectively. The results showed that pressure and the reciprocal value of the ring-down parameters have a favorable linear relationship within their respective pressure range. The sensitivity of the sensor in the low-pressure area was 0.00213 μs·kPa^−1^, the coefficient of determination *R*^2^ was 0.998, and the sensitivity of the sensor in the high-pressure area was 0.01334 μs·kPa^−1^, the coefficient of determination *R*^2^ was 0.998, which had excellent linearity.

**Figure 12:**
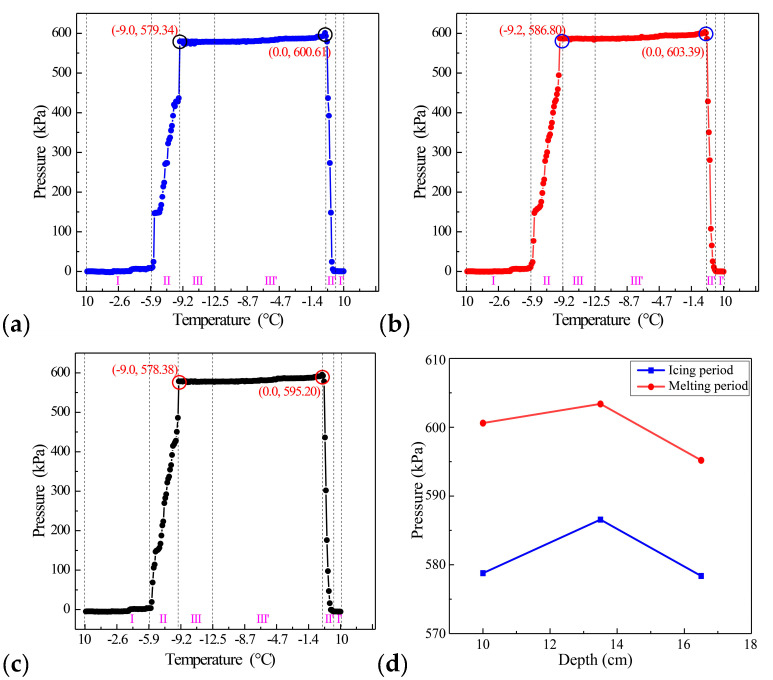
The curve of static ice pressure at different depths; (**a**) sidewall static ice pressure-temperature curve when the depth of the sensor is 10 cm; (**b**) sidewall static ice pressure-temperature curve when the depth of the sensor is 13.5 cm; (**c**) sidewall static ice pressure-temperature curve when the depth of the sensor is 16.5 cm; (**d**) variation curve of static ice pressure along with the depth during the ice growth and melting processes.

When the depths were 10 cm, 13.5 cm, and 16.5 cm, the change of static ice pressure on the sidewall during the ice growth and melting processes is shown in Figure 12.

The growth process of ice can be divided into three stages: Unfrozen stage (I), frozen stage (II), and stable stage (III). When the temperature was higher than −5.9 ℃, almost no pressure was applied to the optical fiber pressure sensor. Moreover, when the temperature dropped from −5.9 ℃ to −9 ℃, the static ice pressure gradually increased and reached 579.34 kPa, 586.80 kPa, and 578.38 kPa at 10 cm, 13.5 cm, and 16.5 cm, respectively. Since then, the static ice pressure had changed slightly but remained unchanged overall. The ice melting process can also be divided into three stages: The stable stage (III’), the melting stage (II’), and the ice melting to water stage (I’). When the temperature is lower than 0 ℃, due to the effect of temperature rise rate, the static ice pressure increases slowly and reaches the maximum values of 600.61 kPa, 603.39 kPa, and 595.20 kPa at 10 cm, 13.5 cm, and 16.5 cm at 0 ℃, respectively. Moreover, when the temperature was higher than 0 ℃, the static ice pressure gradually decreased with the increase of temperature, and no pressure was applied to the optical fiber pressure sensor eventually. As illustrated in Figure 12d, we obtained the curve of the static ice pressure of the sidewall with depth during the ice growth and melting processes. With the increase of depth, the static ice pressure will increase first and then decrease [45]. Besides, the melting pressure was higher than the growth pressure, which may be due to the expansion of the ice layer during the melting process.

### 4.3. Static Ice pressure to the Bottom of the Ice Tank

**Figure 13:**
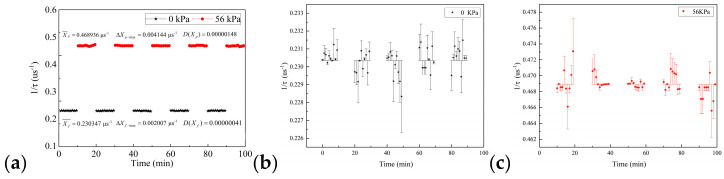
Repeatability test results of pressure sensor on the bottom; (**a**) the ring-down time curve when 56 kPa is loaded and not loaded on the sensor; (**b**) the error bar of 1/*τ* response when 56 kPa is not loaded on the sensor; (**c**) the error bar of 1/*τ* response when 56 kPa is loaded on the sensor.

When the pressure of 56 kPa was loaded and unloaded on the sensor, the repeatability of the bottom static ice pressure was studied by measuring the 1/*τ* response of the sensor, which is shown in Figure 13.

The experimental results showed that when the force was not applied, the average value of the reciprocal of the ring-down parameter was 0.230347 μs^−1^, whereas the maximum error was 0.002007 μs^−1^, and the variance was 0.00000041, as shown in Figure 13b. As shown in Figure 13c, when the force of 56 kPa was applied, the average value of the reciprocal was 0.468936 μs^−1^, the maximum error was 0.004144 μs^−1^, and the variance was 0.00000148. In the experiment, the maximum error was tiny, and the result of variance showed that the data did not fluctuate much, which indicated that the optical fiber sensor had excellent repeatability and could meet the measurement requirements.

**Figure 14:**
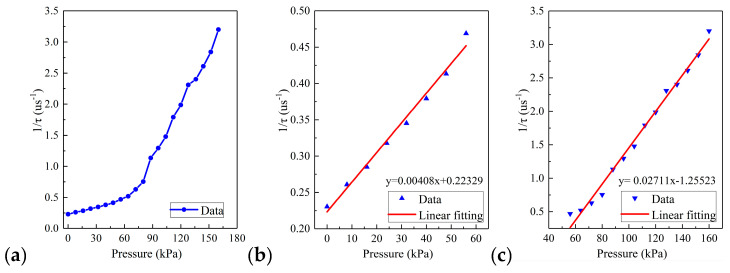
The relationship between the ring-down time and static ice pressure on the bottom; (**a**) ring-down time-pressure curve of the overall range of sensor; (**b**) calibration curve of the low-pressure area; (**c**) calibration curve of the high-pressure area.

The relationship between pressure and ring-down time was demonstrated with continuous pressures varying from 0 kPa to 160 kPa at steps of 8 kPa, which can be seen in Figure 14a.

We can find that when the pressure was 56 kPa, the change rate of the ring-down time changed. Therefore, in the calibration, the pressure was calibrated by segmented calibration with 56 kPa as the boundary. In Figure 14b, the sensitivity of the low-pressure region was 0.00408 μs·kPa^−1^, the coefficient of determination *R*^2^ was 0.978. In addition, in Figure 14c, the sensitivity of the high-pressure region was 0.02711 μs·kPa^−1^; the coefficient of determination *R*^2^ was 0.989. The results indicated that there was a favorable linear relationship between the pressure and the reciprocal of the ring-down time.

**Figure 15:**
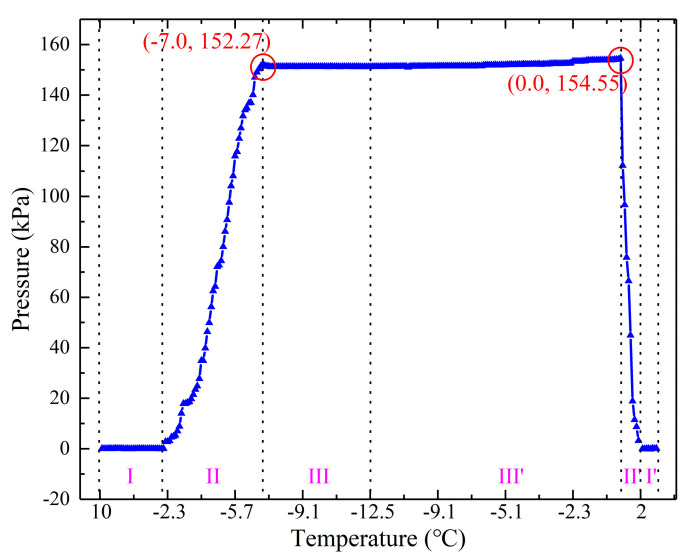
The curve of static ice pressure on the bottom in the growth and melting processes of ice.

The variation of static ice pressure on the bottom during the ice growth and melting processes are shown in Figure 15.

In the unfrozen stage (I), the temperature dropped from 10 ℃ to −2.1 ℃. The water exchanged heat with the experimental environment and began to freeze. In the frozen stage (II), the temperature changed from −2.1 ℃ to −7.0 ℃. The ice started to grow, and the static ice pressure on the bottom increased continuously, reaching the maximum at −7.0 ℃ (152.27 kPa). In the stable stage (III), the ice pressure dropped slightly because the sensor was placed in the open PVC pipe. When the ice entered the growth period, there was pressure on the pressure sensor. The sensor also had a slight upward force acting on the ice, which will push the ice to produce a tiny displacement, and then the pressure will decrease. As the temperature decreased to −12.5 ℃, the pressure tended to be stable. Then we analyzed the pressure variation of ice during the melting stage. In the stable stage (III’), the PVC pipe with the sensor was exposed to the environment of 19.8 ℃, and the ice began to melt. Due to the influence of the temperature rise rate, the static ice pressure began to increase, and the pressure increased to the maximum (154.55 kPa) at about 0 ℃. In the melting stage (II’), the ice began to accelerate melting, and the static ice pressure decreased to 0 kPa rapidly. In the last stage, the ice was melting to water (I’), and there was no static ice pressure at this time.

From these two measurement results, we found that the static ice pressure on the sidewall at 10 cm (579.34 kPa), 13.5 cm (586.80 kPa), and 16.5 cm (578.38 kPa) was higher than that on the bottom (152.27 kPa) in the freezing stage. Similarly, during the melting stage, the static ice pressure on the sidewall at 10 cm (600.61 kPa), 13.5 cm (603.39 kPa), and 16.5 cm (595.20 kPa) was higher than that on the bottom (154.55 kPa). This was because the lateral expansion of the ice layer was constrained by the PVC pipe, while the longitudinal expansion was not affected by the constraint.

## 5. Conclusions

This work constructed a real-time detection system for detecting static ice pressure by combining FPGA’s pulse modulation technology and FLRDS technology. We proposed an FPGA-based optical pulse generation scheme, which realized the generation of pulses with adjustable pulse width and period and reduced the system cost. The optical pulse was applied to the static ice pressure detection system and completed the static ice pressure detection experiment of the bottom and sidewall at different depths. The results show that the system had excellent stability with a variance of 0.00000307. In the static ice pressure experiment on the sidewall, the sensitivity of the system in the low-pressure area and the high-pressure area could reach 0.00213 μs·kPa^−1^ and 0.01334 μs·kPa^−1^, respectively. In the static ice pressure experiment at the bottom, the sensitivity of the system in the low-pressure and high-pressure areas could reach 0.00408 μs·kPa^−1^ and 0.02711 μs·kPa^−1^, respectively. In the experiment, we found that the static ice pressure on the sidewall at different depths was different. As the depth gradually increased, it showed a trend of increasing first and then decreasing, which was roughly consistent with previous research. At the same time, the static ice pressure at the bottom is lower than that at the sidewall, which is because the lateral ice expansion is restrained by the PVC pipe. To summarize, the above report clearly shows that the system can measure the static ice pressure change in real-time during the entire growth and melting processes of the ice, which is of important significance for the continuous real-time detection of static ice pressure on site.
